# Preventive beneficial effects of cannabidiol in a reserpine-induced progressive model of parkinsonism

**DOI:** 10.3389/fphar.2025.1539783

**Published:** 2025-05-08

**Authors:** Alvaro C. Lima, Vinicius S. Bioni, Marcela S. Becegato, Ywlliane Meier, Débora M. G. Cunha, Natan A. Aguiar, Narriman Gonçalves, Fernanda F. Peres, Antônio W. Zuardi, Jaime E. C. Hallak, José A. Crippa, Soraya S. Smaili, Vanessa C. Abilio, Regina H. Silva

**Affiliations:** ^1^ Department of Pharmacology, Universidade Federal de São Paulo, São Paulo, Brazil; ^2^ National Institute for Translational Medicine, National Council for Scientific and Technological Development, Ribeirão Preto, Brazil; ^3^ Department of Neuroscience and Behavior, University of São Paulo, Ribeirão Preto, Brazil; ^4^ Laboratory of Psychiatric Neuroimaging (LIM21), Hospital das Clinicas HCFMUSP, Faculdade de Medicina, Universidade de Sao Paulo, Sao Paulo, Brazil

**Keywords:** Parkinson’s disease, cannabis, animal model, catalepsy, oral movements, tyrosine hydroxylase

## Abstract

**Introduction:**

Parkinson's disease (PD) is characterized by motor and non-motor symptoms such as tremors, difficulty in initiating movements, depression, and cognitive deficits. The pathophysiology of PD involves a gradual decrease in dopaminergic neurons in the substantia nigra, increased inflammatory parameters, and augmented oxidative stress in this region. Several new therapies aim to promote antioxidant and anti-inflammatory actions, including the use of cannabinoids, particularly cannabidiol (CBD). CBD is a non-psychotomimetic component of *Cannabis sativa* that acts broadly through several mechanisms.

**Objective:**

The objective of this study was to investigate the potential protective effect of CBD in mice subjected to a low-dose (0.1 mg/kg) repeated reserpine protocol, which encompasses behavioral and neuronal alterations compatible with the progressiveness of PD alterations.

**Materials and Methods:**

We used two approaches: (1) concurrent administration during the development of parkinsonism and (2) pre-administration to explore a possible preventive action. The effect of CBD (0.5 mg/kg) on reserpine-induced alterations was investigated on behavioral (catalepsy and vacuous chewing movements) and neuronal (immunolabeling for tyrosine hydroxylase - TH) parameters.

**Results:**

Overall, groups that were treated with CBD and reserpine presented motor alterations later during the protocol compared to the groups that received only reserpine (except for vacuous chewing evaluation in the concomitant treatment). Additionally, CBD attenuated reserpine-induced catalepsy (preventive treatment) and prevented the decrease in TH labeling in the substantia nigra pars compacta in both concurrent and preventive protocols.

**Conclusion:**

Based on these data, we observed a beneficial effect of CBD in motor and neuronal alterations reserpine-induced progressive parkinsonism, particularly after preventive treatment.

## 1 Introduction

Parkinson’s disease (PD) is the second most prevalent neurodegenerative disorder, affecting approximately 1% of individuals aged 60 and above (Silva et al., 2024). The disease is characterized by motor impairments such as resting tremor, postural instability, akinesia, and bradykinesia. Additionally, non-motor symptoms are quite common in PD, including depression, cognitive deficits, sleep disorders, among others (Agnello et al., 2024).

PD is a progressive and chronic condition with an unclear etiology. Nevertheless, many factors are known to influence its development, such as environmental features (pesticides, air pollution, and others), lifestyle, and genetic alterations (Silva et al., 2024; Ramirez-Mendoza et al., 2024). These factors could increase oxidative stress and neuroinflammatory activity, which are related to the neurodegeneration process (Cunha et al., 2022; Cohen et al., 2024; Qiao et al., 2024).

Up to date, there is no effective treatment to interrupt the neurodegeneration, although alternatives have been studied to prevent or reduce the progression of PD. In recent years, studies with plant extracts, particularly those with anti-inflammatory and/or antioxidant properties, has increased (Costa et al., 2022; Urbi et al., 2022). One of these possibilities is the use of cannabidiol (CBD) [see Peres, Lima (Peres et al., 2018)].

Two components of approximately 500 components of *Cannabis sativa* are the most studied: CBD and *Δ9-Tetrahydrocannabinol* (THC). While THC induces psychotomimetic effects, CBD does not show this action (Crocq, 2020; Pagano et al., 2022). In addition, there is evidence that CBD can reduce neuroinflammation, oxidative stress, and promote neuroprotection (Muhammad et al., 2022; Booz, 2011; Chen et al., 2016). Thus, the use of a compound that does not promote psychotomimetic effects and has potential for neuroprotection is a promising possibility (Peres et al., 2018; Duncan et al., 2024).

Treatment with CBD has been shown to improve PD-related alterations in some studies, including cellular models (Santos et al., 2015), animal models (Muhammad et al., 2022; Peres et al., 2016; Patricio et al., 2022; da Cruz Guedes et al., 2023), and human patients (Consroe et al., 1986; O'Sullivan et al., 2023). Importantly, treatment with CBD usually does not show potential side effects (Peres et al., 2018; Omotayo et al., 2024). However, this line of research is still scarce and controversial. A recent systematic review grouped some studies that tested cannabinoids in animal models of parkinsonism. Although most of the studies surveyed reported positive effects, there were descriptions of no effects or worsening of some parameters (Alves et al., 2024; Dos-Santos-Pereira et al., 2016; Celorrio et al., 2017). In addition, a study in humans did not show improvement in the Unified Parkinson Disease Rating Scale (UPDRS) scores and BDNF levels after CBD treatment. However, this same study observed an improvement in the Parkinson’s Disease Questionnaire (PDQ-39), a measurement of quality-of-life (Bougea et al., 2020; Chagas et al., 2014).

PD can be studied by simulating the condition in animal models using various substances, some of which have high toxicity and rapid induction of cell death. In this respect, repeated administration of low doses of reserpine induces a slower progression of PD-related alterations (Leao et al., 2015; Lopes-Silva et al., 2024; Santos et al., 2013). This pharmacological model promotes the gradual development of parkinsonian signs and neuronal features, such as inflammation (Cunha et al., 2022), oxidative stress (Silva-Martins et al., 2021; Beserra-Filho et al., 2022), and others. Because of the slow course, this protocol is more likely to detect long-term effects on disease progression and has been an interesting approach to study potential neuroprotective treatments (Peres et al., 2018; Peres et al., 2016; Silva-Martins et al., 2021; Beserra-Filho et al., 2022; Sarmento-Silva et al., 2014; Brandao et al., 2017; Lins et al., 2018; Beserra-Filho et al., 2019; Custodio-Silva et al., 2024).

The aim of this study is to investigate possible CBD neuroprotective effects in the development of parkinsonism induced by low-dose repeated reserpine, using two approaches: (1): administering CBD concurrently with the development of parkinsonism (concomitant treatment), and (2) using CBD as a preventive treatment before the induction of the parkinsonian alterations (preventive treatment).

## 2 Materials and methods

### 2.1 Animals

Six-month-old male Swiss mice were used in this study (N = 75). The animals were housed in groups of 5 per cage (30 × 42 × 16 cm) under controlled airflow, acoustic isolation, and temperature at 22°C ± 1°C with a 12 h light/12 h dark cycle (light on at 7:30 a.m.). There was free access to water and food. Animals used in this study were handled according to the Brazilian law for animal use in research (Law Number 11,794) and all procedures were approved by the local ethics committee (protocol number 3322080217/2017). One animal was euthanized before the end of the protocol, due to fight injuries.

### 2.2 Drug treatment and general procedures

Reserpine (Res, Sigma Chemical Co., United States) was dissolved in glacial acetic acid (1%) and then diluted to the correct concentration with distilled water. The vehicle solution (Veh) consisted of the same amount of acetic acid and water as in the reserpine solution. Animals received subcutaneous injections of Veh or 0.1 mg/kg of reserpine (Res) at a volume of 10 mL/kg body weight.

Purified cannabidiol (CBD - BSPG-Pharm, Sandwich UK) was daily prepared, diluted in 1% tween 80 and saline and administered at dose of 0.5 mg/kg. This dose was chosen based on a previous study of our group conducted with an acute reserpine protocol (Peres et al., 2016). CBD solution and the respective vehicle (saline + tween 80) were administered intraperitoneally at a volume of 10 mL/kg of the animal’s weight.

**FIGURE 1:**
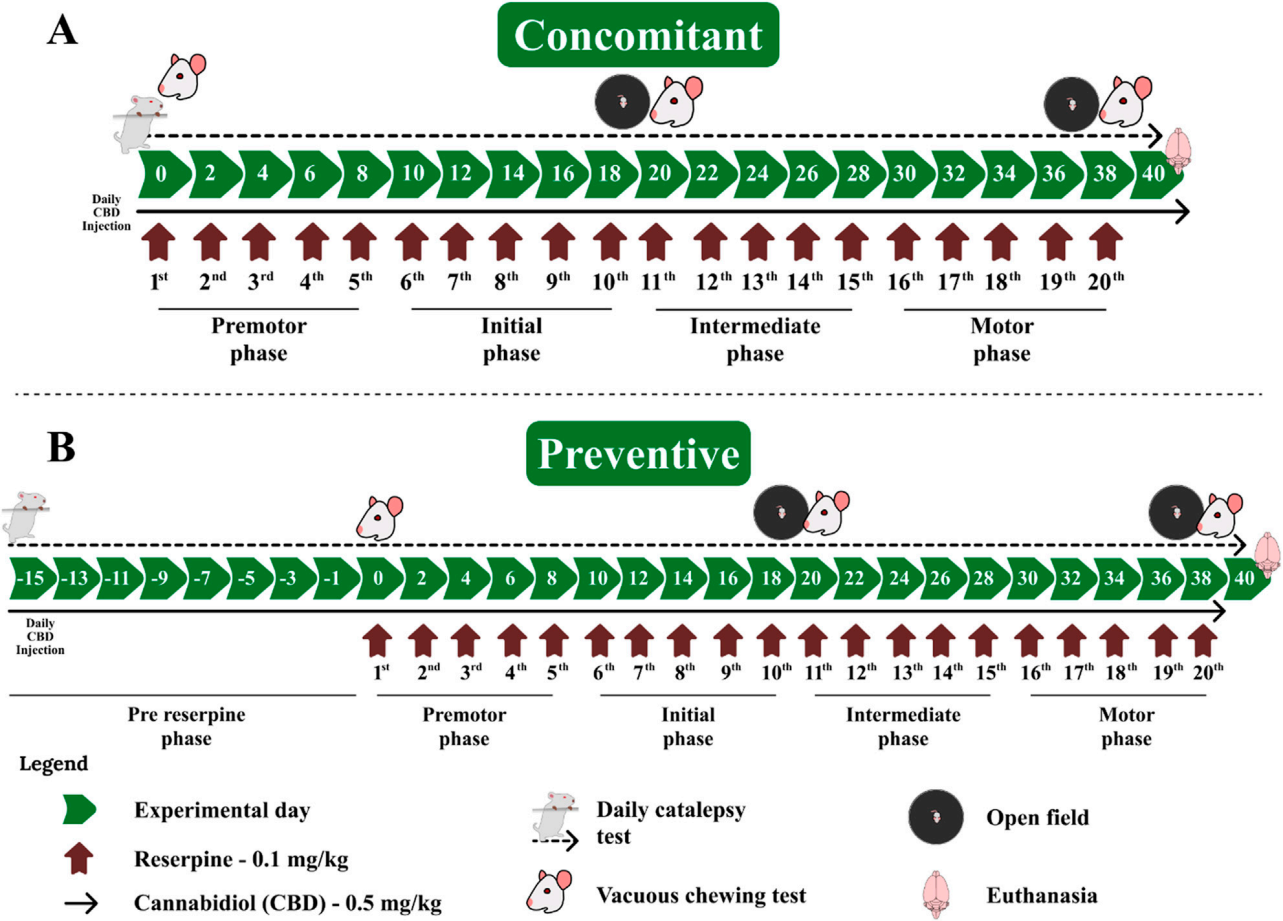
Experimental design of cannabidiol administration **(A)** concomitant and **(B)** preventive protocols relative to repeated reserpine-induced parkinsonism. The green arrows correspond to experimental days of the protocol; the dark red arrows correspond to alternate day administration of reserpine; the continuous black arrows correspond to daily CBD administration; and the dashed black arrow corresponds to daily catalepsy tests.

The CBD/saline treatment was administered daily between 10 a.m. and 12 p.m. The reserpine/vehicle treatment occurred on alternate days, 30 min after the administration of CBD or saline. Across the treatment, animals were submitted to the following procedures: (1): catalepsy test (before the first injection and daily across treatment); (2); open field 48 h after the 10th and 20th injection of reserpine, and (3) oral movements after the open field evaluations. All behavioral tests were conducted prior to any drug administration. The experimental design is shown in Figure 1.

**FIGURE 2:**
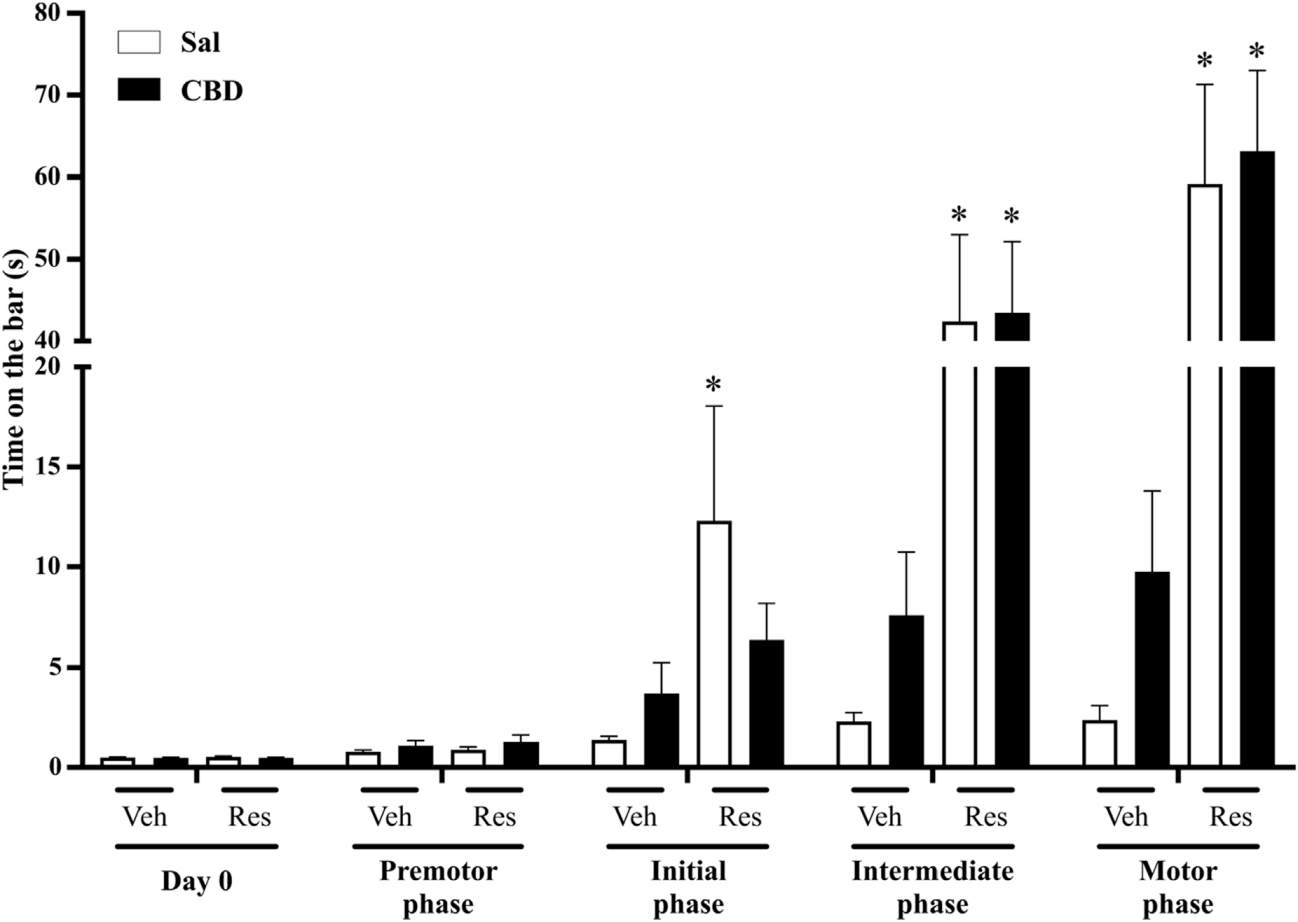
Catalepsy phases across the concomitant protocol. Each phase represents the mean of the assessments conducted under 5 reserpine applications: Day 0 (previously to reserpine injection), premotor (1^st^ to 10^th^ assessments), initial (11^th^ to 20^th^ assessments), intermediate (21^st^ to 30^th^ assessments), and motor (31^st^ to 40^th^ assessments). Individual animal data in Supplementary Material S1. * Difference between vehicle (Veh) and reserpine (Res) groups treated concomitantly with saline (Sal) or cannabidiol (CBD) (ANOVA with repeated measures and Sidak’s test, p ≤ 0.05). Data are expressed as means ± SEM (*n = 10*).

We conducted two different protocols (1) the administration of CBD starting with the reserpine protocol (concomitant protocol - Figure 1A) and (2) initiating the administration of CBD 2 weeks before the beginning of the reserpine protocol (preventive protocol - Figure 1B).

### 2.3 Behavioral evaluation

#### 2.3.1 Catalepsy test

The catalepsy behavior was evaluated by placing the animal’s forepaws on a horizontal bar positioned 5 cm above the bench surface, while the hind paws rested on the bench. Once placed in this position by the experimenter, the animals were allowed to move freely. The time taken for the animal to withdraw one or both forepaws from the bar was measured, with a maximum trial duration of 180 s. Three trials were conducted for each animal on every observation day, and the results were analyzed based on the mean value of these trials. This behavior is characterized by the incapacity of the animal to change position, and it has been associated with decreased motor function, such as akinesia and bradykinesia (Sanberg et al., 1988; Gerlach and Riederer, 1996; Gobira et al., 2013).

The analysis was conducted in phases, each comprising 10 observation days (corresponding to 5 reserpine injections), except for the basal phase, which comprised 1 observation day: day 0 (basal phase, no injection administered), premotor phase (1^st^ to 5^th^ injection), initial phase (6^th^ to 10th injection), intermediate phase (11^th^ to 15^th^ injection), and motor phase (16^th^ to 20^th^ injection). The division of the phases was determined considering equal distribution of number of reserpine injections and the profile of catalepsy intensity, as conducted in previous studies of our group (Lima et al., 2021).

#### 2.3.2 Vacuous chewing test

The animals were placed individually in a wire cage (20 × 20 × 15 cm). Mirrors were positioned beneath and behind the cage to enable behavioral observations when the animal was facing away from the observer. The frequency of vacuous chewing movements (defined as mouth openings in the vertical plane not directed towards physical material) was continuously observed for 10 min. This behavior is characterized by dysfunctional balance between neurotransmitters involved in motor control and is associated with resting tremors in PD (Pirker et al., 2023; Salamone et al., 1998).

#### 2.3.3 Open field test

The open field (OF) was performed in a cylindrical arena of opaque white polyethylene with a black-painted wooden base (50 cm high walls and 40 cm diameter base). A camera was positioned above the apparatus to record the sessions. was used. Each animal was individually evaluated for 5 min and the distance traveled in the apparatus (m) was registered by an animal video-tracking software (Anymaze, Stoelting, United States). The apparatus was cleaned with a 5% ethanol solution between tests.

### 2.4 Tissue processing and tyrosine hydroxylase immunohistochemistry

At the end of the experiments, the animals were pre-anesthetized with 0.5 mg/kg of Fentanyl and 1 mg/kg of Acepromazine intramuscularly (IM), as per veterinary guidance. After 5 min, Ketamine hydrochloride and Xylazine hydrochloride were administered intraperitoneally at doses of 100 mg/kg and 10 mg/kg, respectively, at volumes of 10 mL/kg.

Once the animals were deeply anesthetized, they were euthanized by transcardiac perfusion using a 0.1 M phosphate buffer solution with pH 7.4% and 4% paraformaldehyde. After craniotomy, the brains were removed and immersed in 4% paraformaldehyde for 24 h at 4°C. After 24 h, the brains were transferred to a 30% sucrose solution at 4°C. Each brain was embedded in Tissue-Tek^®^ (Sakura, Japan) and frozen at −20°C. Subsequently, the brains were sliced serially in the coronal plane into 40 μm thick sections using a cryostat microtome (Leica, Germany) at −20°C. The sliced sections were stored in an antifreeze solution at −20°C.

The tissue samples underwent the immunohistochemical process using the free-floating protocol. Sections were washed four times in PBS for 5 min (repeated at each protocol step), followed by a wash with 0.03% hydrogen peroxide for 20 min. For tyrosine hydroxylase (TH) detection, sections were incubated with a polyclonal anti-TH antibody produced in rabbit (Millipore, USA, 1:3,000) diluted in 0.4% Triton X-100 and PBS with 2% albumin for 24 h at 4°C.

Afterwards, the sections were incubated with biotinylated IgG anti-rabbit antibody (Vector Labs, United States, 1:500) diluted in 0.4% Triton X-100 and PBS for 2 h at 4°C. Following this, the tissue samples were washed with PBS and incubated with avidin-biotin-peroxidase solution (ABC Elite Kit, Vector Labs, Burlingame, United States, 1:500) diluted in 0.4% Triton X-100 with NaCl and PBS for an additional 2 h.

Finally, the reaction was started by adding 3,3-diaminobenzidine (DAB-Sigma, Aldrich, United States) and 0.01% hydrogen peroxide in 0.1M PBS. After tissue labeling, the sections were mounted on gelatinized histological slides and analyzed under a microscope (Nikon Eclipse 80i) coupled with a camera (MBF biosciences, United States). Images were obtained from approximately 12 sections (24 images, from both sides) of the Substantia Nigra pars compacta (SNpc).

To estimate the number of TH+ cells in SNpc, we used ImageJ (NIH, United States) to select each target cell and count them. We analyzed 8–12 sections of each animal [4 – 6 slices equally distributed from −2.70 mm to −3.52 mm from bregma, according to Paxinos and Franklin (Paxinos and Franklin, 2004)]. The means of all measures were calculated, and the data were normalized by the mean value of the control group [mean TH+ cell number of each animal/mean of the TH+ of control group (Veh/Sal)] to evaluate the proportional alterations.

### 2.5 Data analysis

Data normality and homogeneity of variances were tested by Shapiro-Wilk and Levene’s tests, respectively. The catalepsy test and vacuous chewing test were analyzed by two-way repeated measures ANOVA followed by Sidak’s *post hoc*. Nonparametric data from TH+ cells quantification was analyzed by Mann-Whitney U-test in each group separately.

Results were expressed as mean ± SEM (parametric analyses–behavioral analyzes), and median with maximum and minimum values (nonparametric analyses–TH+ cell immunostaining), and p ≤ 0.05 were considered to reflect significant differences. In addition, size effect was calculated (η^2^ partial (η^2^p) for parametric analyses, and “r” for non-parametric analyses). All statistical analysis was made using SPSS (IBM, USA) and the graphics were produced using Prism (GraphPad, USA).

## 3 Results

### 3.1 Concomitant administration of CBD with reserpine protocol

#### 3.1.1 Catalepsy test

This evaluation aimed to investigate the effects of CBD on the latency to initiate movement. The test consists of placing the front paws in the bar and quantifying the latency for the animal to remove one or both paws from the bar. Two-way repeated measures ANOVA showed significant effects of time [F (4,132) = 44.255, p < 0.001, η^2^p = 0.573], reserpine treatment [F (1,33) = 28.723, p < 0.001, η^2^p = 0.465] and the interaction between time and reserpine treatment [F (4,132) = 31.187, p < 0.001, η^2^p = 0.486]. The Sidak’s *post hoc* test demonstrated increased values in reserpine compared to vehicle groups, which received saline, at the initial, intermediate, and motor phases (p = 0.038, 0.001 and p < 0.001, respectively). Conversely, reserpine animals that received concomitant CBD showed increased catalepsy compared to respective control only at the intermediate and motor phases (p = 0.002 and p < 0.001, respectively), demonstrating a delay on the motor deficit onset. However, we did not observe a significant difference between Sal and CBD in the reserpine animals, as shown in Figure 2 (individual data is shown in Supplementary Material S1).

#### 3.1.2 Vacuous chewing test

**FIGURE 3:**
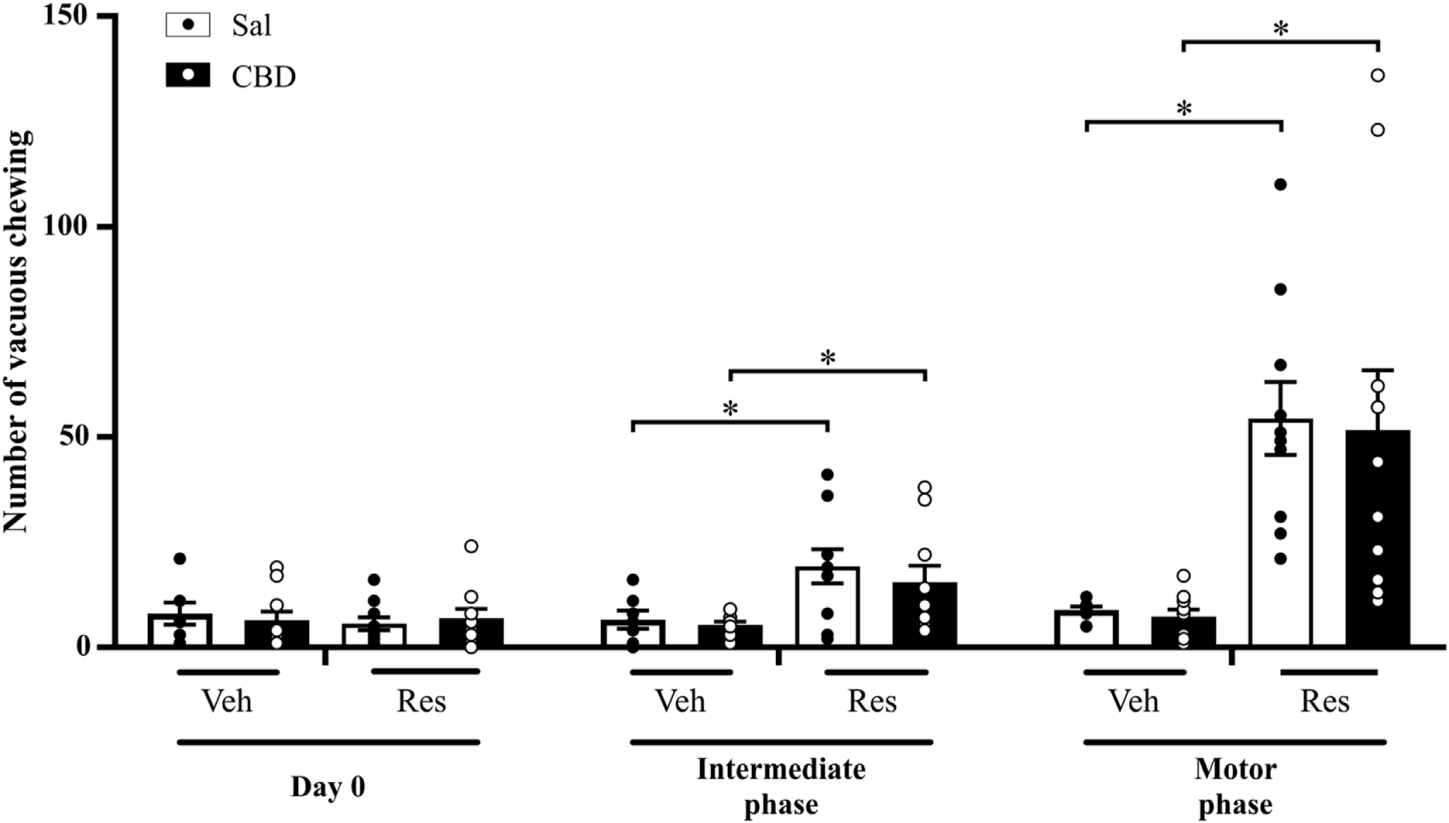
Number of vacuous chewing across the concomitant protocol. Circles indicate individual data in each group. *Difference between vehicle (Veh) and reserpine (Res) groups treated concomitantly with saline (Sal) or cannabidiol (CBD) (ANOVA with repeated measures and Sidak’s test, p ≤ 0.05). Data are expressed as means ± SEM (*n = 10*).

This evaluation aimed to investigate the effects of CBD on oral dyskinesia. The test consists of placing the animal in a cage and quantifying the purposeless mandibular movements. Two-way repeated measures ANOVA showed significant effects of time [F (2,66) = 20.070, p < 0.001, η^2^p = 0.378], reserpine treatment [F (1,33) = 28.503, p < 0.001, η^2^p = 0.463], and the interaction between time and reserpine treatment [F (2,66) = 17.879, p < 0.001, η^2^p = 0.351]. The effect of CBD on the reserpine-induced alterations was not observed. The Sidak’s *post hoc* test demonstrated that vacuous chewing was increased in both reserpine groups compared to respective control groups at the intermediate phase (Sal: p = 0.024, and CBD: p = 0.012), and the motor phase (Sal: p = 0.001, and CBD: p = 0.002), as shown in Figure 3.

#### 3.1.3 Open field test

This evaluation aimed to observe the effect of CBD on locomotion in the OF. Two-way repeated measures ANOVA showed significant effects of time [F (1,33) = 67.286; p < 0.001, η^2^p = 0.671], reserpine treatment [F (1,33) = 124.324; p < 0.001, η^2^p = 0.790], and CBD [F (1,33) = 6.603; p < 0.015, η^2^p = 0.15]. However, an effect of CBD in the reserpine induced locomotion impairment was not observed in the pos hoc analysis. Indeed, Sidak’s *post hoc* test revealed a significant effect of reserpine decreasing the locomotion in the intermediate (Sal: p < 0.001, and CBD: p < 0.001) and motor (Sal: p < 0.001, and CBD: p < 0.001) phases. The only CBD effect observed was an increase in locomotion in Veh/CBD compared to Veh/SAL (p = 0.016), as shown in Table 1.

**TABLE 1:** Distance traveled in the open field (m) of concomitant and preventive protocols, at the intermediate phase (48 h after 10^th^ reserpine injection), and motor phase (48 h after 20^th^ reserpine injection). Data are expressed as means ± SEM *(n = 9–10).*

		Concomitant protocol		Preventive protocol	
		Intermediate phase	Motor phase	Intermediate phase	Motor phase
Veh	Sal	15.795 ± 0.786	10.467 ± 0.786	11.643 ± 0.918	10.964 ± 1.235
	CBD	20.158 ± 1.335	11.956 ± 1.437^#^	12.218 ± 1.186	12.073 ± 1.794
Res	Sal	6.884 ± 1.236*	2.525 ± 1.070	8.389 ± 1.220*	7.027 ± 1.464*
	CBD	9.391 ± 0.902*	2.669 ± 0.592	8.452 ± 1.115*	6.000 ± 0.082*

*Difference between Veh-Res.

#Difference between Sal-CBD (p < 0.05).

#### 3.1.4 Immunohistochemistry

**FIGURE 4:**
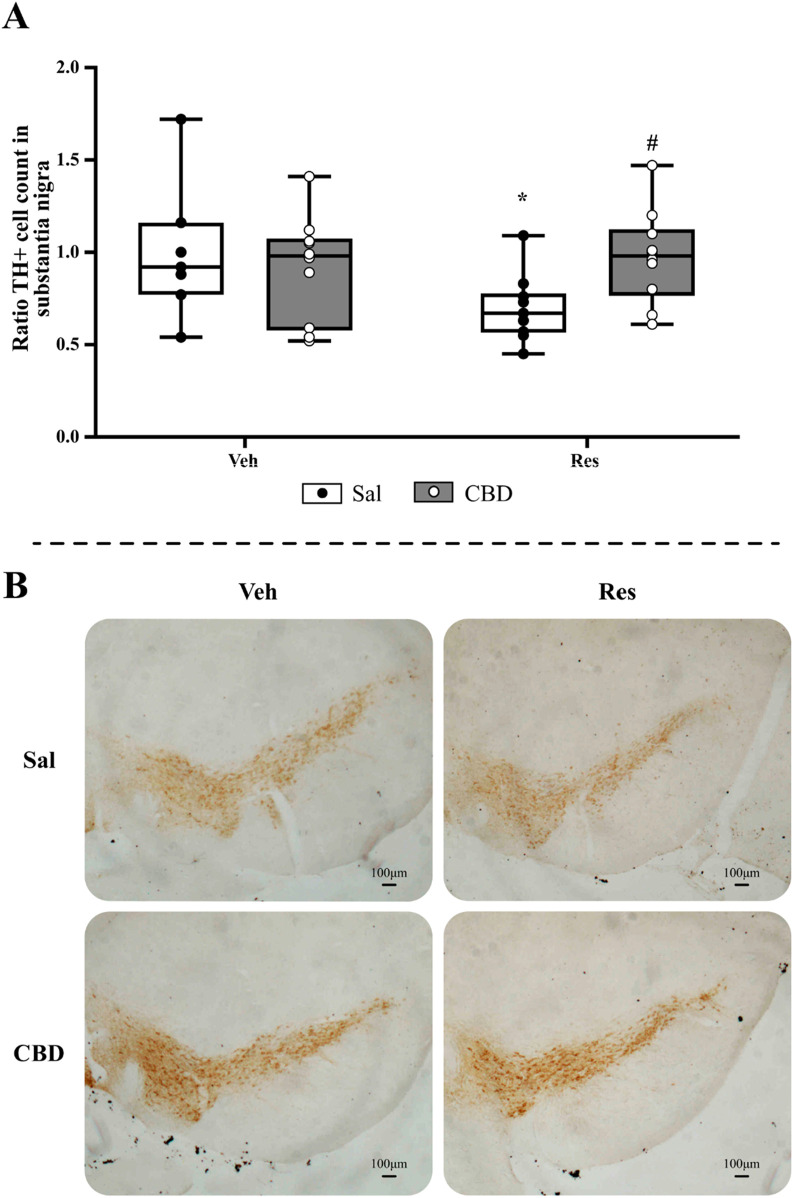
Immunohistochemical analyses of the concomitant protocol, including **(A)** TH+ cell count in the substantia nigra pars compacta, and **(B)** representative images from SNpc of each group (objective ×5). Circles indicate individual data in each group. *Difference between vehicle (Veh) and reserpine (Res), ^#^Differences between saline (Sal) and cannabidiol (CBD) (Mann-Whitney test; p ≤ 0.05). Data are expressed as median with max and minimum values (*n = 9–10*), scale bar: 100 µm.

This evaluation aimed to investigate the effect of CBD on the status of functioning dopaminergic neurons by immunostaining of TH+ cells. The analysis of TH+ cell quantification in the SNpc (Figure 4A) by the Mann-Whitney test showed significant reduction in cell count of reserpine/saline group compared to vehicle/saline (U = 14.000, p = 0.040, r = 0.51). Furthermore, the reserpine group that received CBD showed an increased value compared to reserpine/saline group (U = 19.000, p = 0.019, r = 0.53). Representative images of each treatment group are shown in Figure 4B.

### 3.2 Preventive administration of CBD with reserpine protocol

#### 3.2.1 Catalepsy test

**FIGURE 5:**
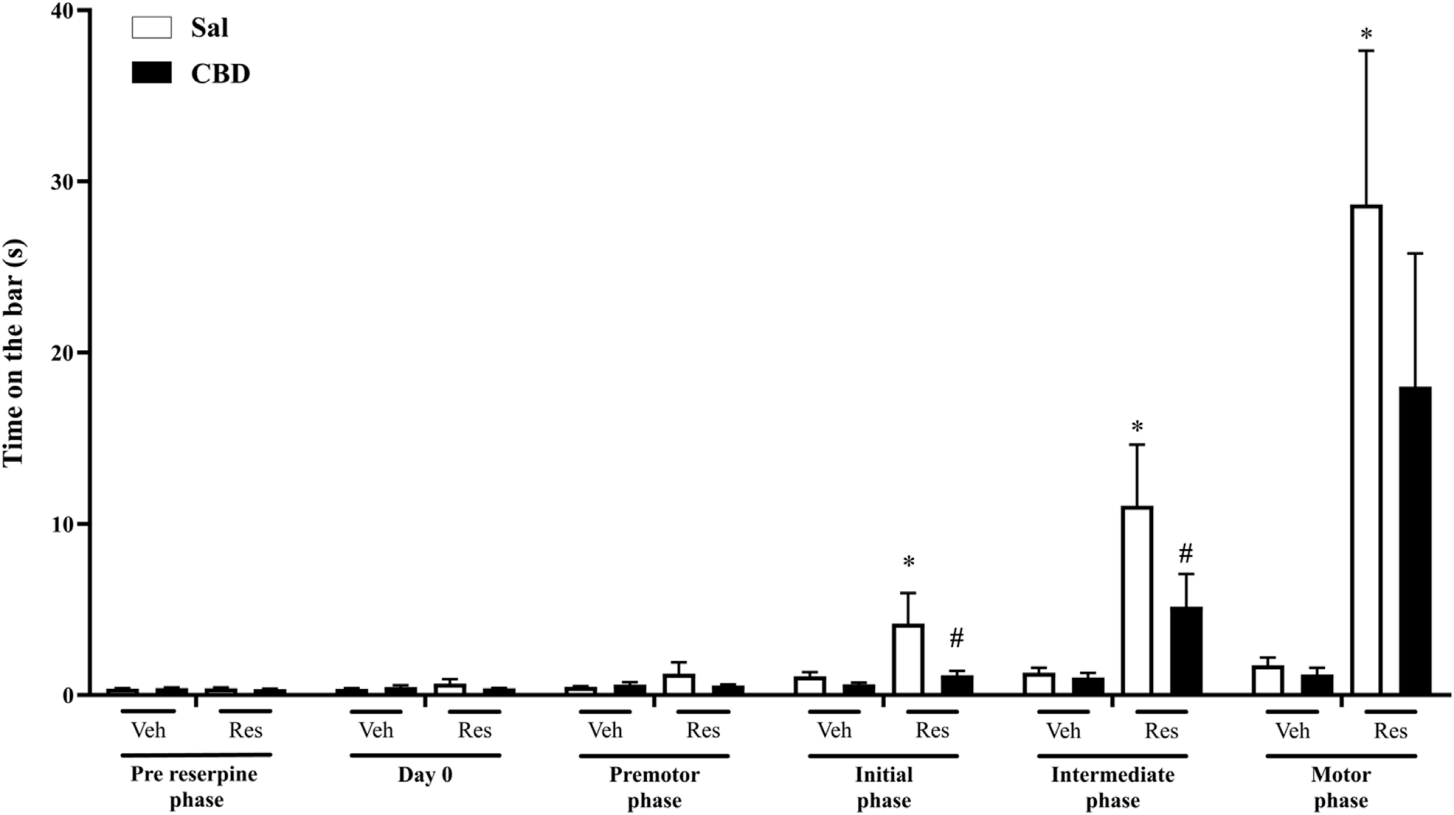
Catalepsy phases across the preventive protocol, each phase represents the mean of 5 reserpine applications: pre reserpine and day 0 (previously to reserpine administration), premotor (1^st^ to 10^th^ assessments), initial (11^th^ to 20^th^ assessments), intermediate (21^st^ to 30^th^ assessments), and motor (31^st^ to 40^th^ assessments). Individual data in Supplementary Material 2. *Difference between vehicle (Veh) and reserpine (Res) groups (ANOVA with repeated measures and Sidak’s test, p ≤ 0.05), ^#^Difference between saline (Sal) and cannabidiol (CBD). Data are expressed as means ± SEM (*n = 9–10*).

The daily administration of preventive CBD promoted a significant reduction in the latency for the animals to start the movement and leave the bar. Two-way repeated measures ANOVA showed significant effects of time of administration [F (5,170) = 13.835, p = 0.001, η^2^p = 0.289], reserpine treatment [F (1,33) = 11.860, p = 0.002, η^2^p = 0.259] and the interaction between reserpine and time of administration [F (5,170) = 11.604, p = 0.001, η^2^p = 0.254], as shown in Figure 5 (individual data is shown in Supplementary Material S2). Sidak’s *post hoc* revealed a significant increase in catalepsy behavior in the reserpine group that receive saline, in initial, intermediate, and motor phases (p = 0.03, 0.003, and 0.005, respectively). Furthermore, it is shown that the Res/CBD group has ameliorated (decreased) the catalepsy behavior, when compared to Res/Sal, in the initial (p = 0.029) and intermediate phases (p = 0.05).

#### 3.2.2 Vacuous chewing test

**FIGURE 6:**
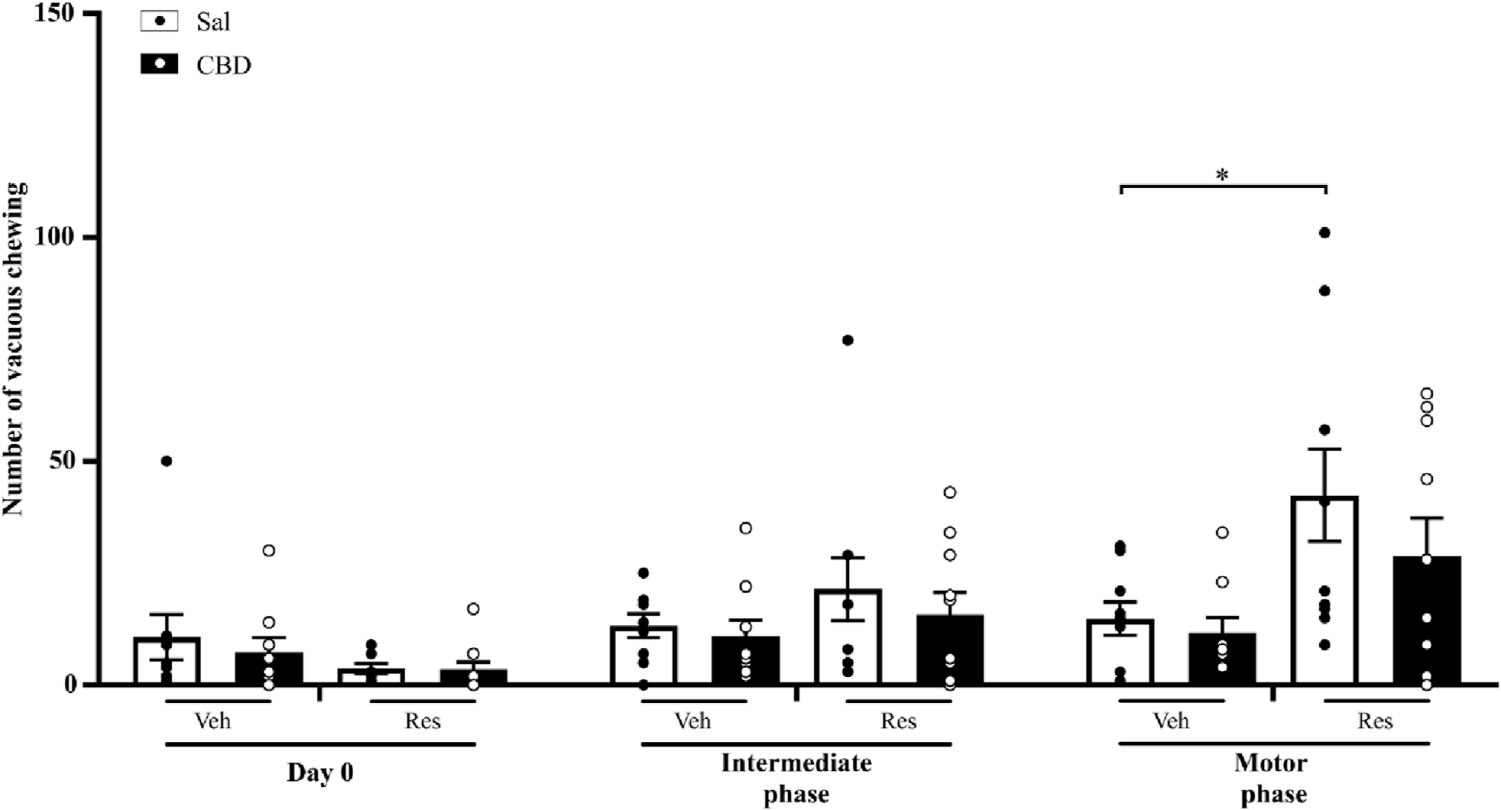
Number of vacuous chewing across the preventive protocol. Circles indicate individual data in each group. *Difference between vehicle (Veh) and reserpine (Res) groups treated concomitantly with saline (Sal) or cannabidiol (CBD) groups (ANOVA with repeated measures and Sidak’s test, p ≤ 0.05). Data are expressed as means ± SEM (*n = 9–10*).

The preventive CBD administration protected the animals from the impairment promoted by reserpine, preventing involuntary mandibular movements. Two-way repeated measures ANOVA reveal significant effects of time of administration [F (2,68) = 17.772, p < 0.001, η^2^p = 0.343] and the interaction between reserpine treatment and time of administration [F (2,68) = 10.523, p < 0.001, η^2^p = 0.236]. Sidak’s *post hoc* showed an increase in vacuous chewing quantity only in reserpine saline group, in the motor phase (p = 0.013), demonstrating CBD effect prevented the motor impairment. Nevertheless, we did not observe a significant difference between Sal and CBD groups that received reserpine at the motor phase, as shown in Figure 6.

#### 3.2.3 Open field test

Along the protocol the animals were exposed to three moments, at Day 0 (without reserpine administration), at intermediate phase (after 10 reserpine injections), and motor phase (after 20 reserpine injections). Two-way repeated measures ANOVA revealed only effect of reserpine [F (1,34) = 18.369; p < 0.001, η^2^p = 0.351]. Sidak’s *post hoc* test showed a decrease in locomotion in reserpine animals in intermediate (Sal: p = 0.048; CBD: p = 0.031) and motor (Sal: p = 0.05; CBD: p = 0.002) phases. We did not observe any significant effect of CBD on the reserpine animals, as shown in Table 1.

#### 3.2.4 Immunohistochemistry

**FIGURE 7:**
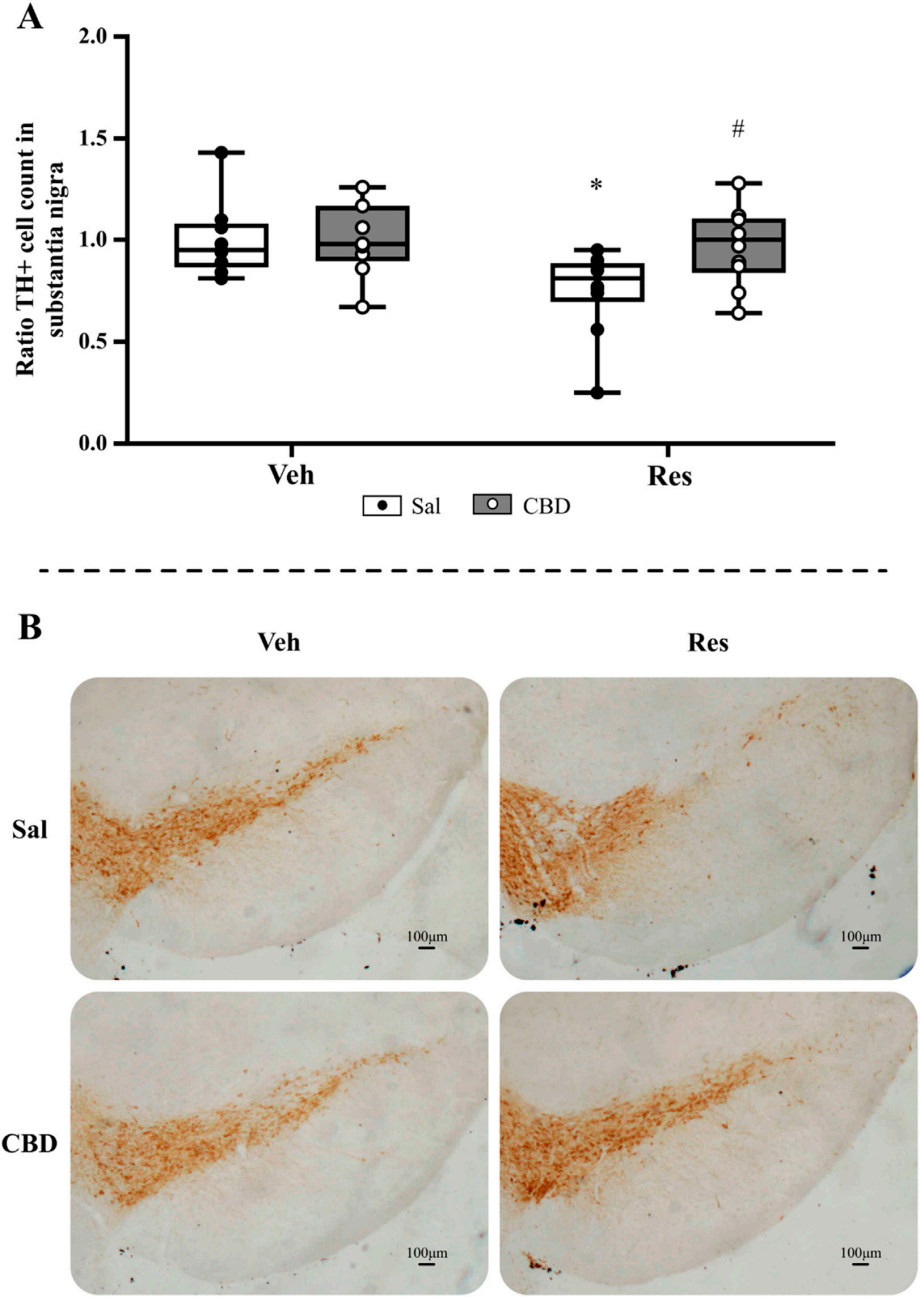
Immunohistochemical analyses of preventive protocol, including **(A)** TH+ cell count in the substantia nigra pars compacta, and **(B)** representative images from SNpc of each group (objective ×5). Circles indicate individual data in each group. *Difference between vehicle (Veh) and reserpine (Res), ^#^Differences between saline (Sal) and cannabidiol (CBD) (Mann-Whitney test; p ≤ 0.05). Data are expressed as median with max and minimum values (*n = 9–10*), scale bar: 100 µm.

The TH+ cell quantification analysis in SNpc (Figure 7A) by Mann-Whitney test showed a significant decrease in the cell average in the reserpine group that received saline (U = 13.500, p = 0.01, r = 0.60); and the Res/CBD group showed increased values compared to Res/saline (U = 21.000, p = 0.028, r = 0.50). Representative images of each treatment group are shown in Figure 7B.

## 4 Discussion

This study aimed to investigate a possible protective effect of cannabidiol (CBD) in progressive reserpine-induced model of parkinsonism, using two approaches: (1): concomitant treatment with CBD and reserpine, and (2) pre-treatment with CBD before reserpine administration. Our results showed that both concomitant and pre-treatment with CBD have protective effects on reserpine-induced parkinsonism, as evidenced by improvements in behavior and TH+ cell count.

Parkinson’s disease can be studied in experimental models by different approaches. In rodents, these approaches include, among others, genetic alterations/mutations (*e.g.,* α-synuclein mutation), infusion of neurotoxins (*e.g.,* 6-hydroxydopamine – 6-OHDA, and 1-methyl-4-phenyl-1,2,3,6-tetrahydropyridine–MPTP), and administration of pharmacological agents (*e.g*., reserpine). Each one has limitations and none of them reproduce all the features of the condition in humans. Genetically determined PD (familial form) stands for just 5%–10% of the patients (Dovonou et al., 2023). Although inducing specific dopaminergic degeneration, the use of neurotoxins also has limitations. For example, 6-OHDA is infused unilaterally directly in the brain, promoting a quick degeneration in one of the hemispheres, leading to immediate severe contralateral motor impairment (Leal et al., 2016; Jagmag et al., 2015). In addition, studies have shown that MPTP can produce degeneration without inducing motors impairments (Leal et al., 2016; Jagmag et al., 2015; Imbriani et al., 2022). The acute administration of reserpine rapidly induces severe motor impairment (Carlsson et al., 1957; Lorenc-Koci et al., 1995; Cavalheiro et al., 2022; Volta et al., 2010), but the repeated long-term administration of a lower dose of this drug induces a gradual appearance of such impairment. Nevertheless, unlike the condition in humans, the motor deficit is reversible after treatment withdraw (Santos et al., 2013), although the decreased dopaminergic immunostaining persists (Santos et al., 2013; Melo et al., 2022).

In the present study, we chose to use the chronic protocol with a low dose of reserpine (Silva et al., 2024; Leao et al., 2015; Leal et al., 2016; Fernandes et al., 2012) because the progressiveness of motor alterations is more likely to show long-term effects of potential neuroprotective treatments (Peres et al., 2018; Peres et al., 2016; Silva-Martins et al., 2021; Beserra-Filho et al., 2022; Sarmento-Silva et al., 2014; Brandao et al., 2017; Lins et al., 2018; Beserra-Filho et al., 2019; Custodio-Silva et al., 2024). This protocol produces gradual motor impairments in both rats (Silva et al., 2024; Cunha et al., 2022; Santos et al., 2013; Lima et al., 2021) and mice (Lopes-Silva et al., 2024; Beserra-Filho et al., 2022; Campelo et al., 2017), and rodents submitted to this protocol develop non-motor signs (Santos et al., 2013; Sarmento-Silva et al., 2014; Lima et al., 2021; Campelo et al., 2017), inflammatory reactions (Cunha et al., 2022), oxidative impairment (Silva-Martins et al., 2021; Fernandes et al., 2012; Leao et al., 2017) and neurotransmitter impairments (Cunha et al., 2022; Lopes-Silva et al., 2024; Santos et al., 2013; Lima et al., 2021; Campelo et al., 2017) similar to those observed in PD patients (Silva et al., 2024). Our results showed that the chronic treatment with reserpine promoted a motor impairment, increasing the time at catalepsy bar and the vacuous chewing movements, and decreasing the TH+ cell count, corroborating previous studies in mice (Lopes-Silva et al., 2024; Silva-Martins et al., 2021; Beserra-Filho et al., 2022; Campelo et al., 2017; Soares et al., 2021).

Cataleptic behavior is characterized by an inability to change the position imposed on the animal. An increase in this behavior is associated with a decrease in motor function (Gerlach and Riederer, 1996). Therefore, catalepsy has been related to parkinsonian symptoms, such as bradykinesia (Sanberg et al., 1988; Gerlach and Riederer, 1996). Our results showed that reserpine gradually increased the duration of catalepsy, indicating that the reserpine group developed motor impairment. These findings are consistent with the literature using the same protocol in mice (Lopes-Silva et al., 2024; Beserra-Filho et al., 2022; Campelo et al., 2017; Soares et al., 2021). Additionally, CBD administration was able to significantly delay the development of cataleptic behavior. In the concomitant protocol, this treatment delayed the onset of catalepsy by one phase, while the CBD preventive protocol inhibited the significant increase in catalepsy time in all phases.

Vacuous chewing in rodents reflects an imbalance between dopaminergic and cholinergic neurons and is associated with resting tremors (Salamone et al., 1998). This imbalance can be induced by mechanisms that decrease dopamine activity, such as antipsychotic drugs and other dopamine receptor antagonists (Salamone et al., 1998; Turrone et al., 2002). Reserpine (given acutely) promotes increased oral movements, which could be associated with tardive dyskinesia (Silva et al., 2002). At the same time, in the repeated administration of low doses of reserpine model, vacuous chewing increases progressively throughout the protocol (Beserra-Filho et al., 2022; Lima et al., 2021; Soares et al., 2021). Our results are consistent with these studies, showing that reserpine increases the frequency of vacuous chewing movements. Concomitant CBD did not affect the vacuous chewing increase. However, the significant increase in Res/CBD group was not observed in preventive protocol. There is evidence that resting tremors (which are associated with vacuous chewing evaluation) are related to an impairment in the serotonergic system (Pirker et al., 2023; Loane et al., 2013). In this respect, there is evidence of a partial or allosteric modulation of 5HT-1A receptor activation induced by CBD (Alexander et al., 2025). Furthermore, CBD can block the acetylcholinesterase enzyme, interfering with the imbalance between dopamine e acetylcholine, although this mechanism have been related to the cognitive symptoms in PD (Puopolo et al., 2022). Finaly, we observed that the effect of CBD was more pronounced in the catalepsy evaluation than in the oral movements assessment. Although our data do not provide evidence of the mechanisms involved in this difference, it is important to mention that catalepsy and oral movements are associated with different aspects of motor symptoms in PD, namely, akinesia/bradykinesia and resting tremor (Sanberg et al., 1988; Gobira et al., 2013; Salamone et al., 1998), and different symptoms might respond differently to treatments.

The effects of CBD on parkinsonian-related motor impairments described in the present study corroborate previous findings from our group (Peres et al., 2016) using a higher dose of reserpine (1.0 mg/kg) in an acute treatment (2 injections), in which CBD also promoted beneficial effects on catalepsy and vacuous chewing evaluations. In this previous study, CBD was inefficient in ameliorating the decrease in open-field locomotion. In this respect, we also conducted locomotion evaluation in the open field, and we did not observe any effect of CBD treatment in the reserpine group (as demonstrated in Table 1). Nevertheless, an increase in locomotion in the vehicle-treated CBD group was observed during the first evaluation of this test. Although this result corroborate a previous finding with acute CBD administration (Kasten et al., 2019), some studies did not observe this increase in OF locomotion induced by CBD (Calapai et al., 2022; Zieba et al., 2019). The lack of CBD effect in reserpine groups could be related to the high decrease in motivation to explore the open field combined with the motor impairment promoted by reserpine treatment, as observed by other studies (Silva-Martins et al., 2021; Beserra-Filho et al., 2022; Lins et al., 2018).

Although conducted with similar treatments and behavioral assessments, it is worth highlighting some differences between the aforementioned study and the present one. The acute high-dose reserpine protocol does not consider the progressive development of motor alterations, as in the case of the chronic low-dose scheme. Despite that, the study with the acute protocol did show the beneficial effects of CBD, but to a lesser extent (only on the last observation day of the catalepsy test) and without the evaluation of TH immunostaining. In contrast, the present study showed a new perspective of CBD effect, showing a reduction in the progression of the motor impairment which was associated with a prevention of neuronal damage, in both protocols (concomitant and preventive).

In animal models, the reduced dopaminergic function typical of PD is usually observed by diminished labeling of tyrosine hydroxylase (a rate limiting enzyme in dopamine synthesis). This measure is commonly assessed in different models, such as 6-OHDA (Matheus et al., 2016; Shao et al., 2024), MPTP (Wang et al., 2024), and chronic reserpine protocol (Beserra-Filho et al., 2022; Brandao et al., 2017; Lins et al., 2018; Lima et al., 2021). In accordance, the reserpine-treated group showed a reduction in TH+ cell count in substantia nigra, demonstrating the functional impairment of dopaminergic neurons. Importantly, both the preventive and the concomitant treatment with CBD were able to hinder the reserpine-induced reduction in TH+ cells count.

Among several mechanisms of action of CBD is the reduction of oxidative stress and neuroinflammation. One possible mechanism involved in oxidative stress reduction is the presence of a hydroxy group that acts as an antioxidant agent, thereby reducing ROS in the cytoplasm (Jha et al., 2024). Additionally, CBD increases antioxidant substances such as superoxide dismutase-3, glutathione-s-transferase and catalase expression. There is also evidence that the anti-inflammatory action of this compound is related to the inhibition of protein aggregation, which is strongly linked to the pathogenesis of neurodegenerative processes by inducing reactive inflammation (Jha et al., 2024). Specifically, a study showed that CBD reduced α-synuclein aggregation in a *C. elegans* model (Muhammad et al., 2022). In addition, CBD effects against neuroinflammation could be related to inhibition of MAPK and NFκB phosphorylation (Peres et al., 2018; Jha et al., 2024; Kim et al., 2023), blockage of the inflammasomes activation, reduction of pro-inflammatory cytokines (Chu et al., 2024), interaction with adenosine receptors, PPARγ, among others (Chu et al., 2024; Sunda and Arowolo, 2020). Indeed, as mentioned previously, inflammatory and oxidant factors can play an important role in the PD development (Qiao et al., 2024). Further, non-steroidal anti-inflammatory drugs can reduce the risk and ameliorate the parkinsonian symptoms (Alrouji et al., 2023; Pereira et al., 2021). In this respect, the repeated reserpine protocol induces increases in both oxidative stress and neuroinflammation parameters (Cunha et al., 2022; Lopes-Silva et al., 2024; Silva-Martins et al., 2021). In a previous study using this same protocol, the alcoholic monoterpene myrtenol reduced the oxidative status index in the dorsal striatum, and this effect was accompanied by amelioration of motor function in mice (Silva-Martins et al., 2021).

An important neuroprotective effect of CBD was shown in different parkinsonism rodent animal models induced by 6-OHDA (Garcia et al., 2011), MPTP (Wang et al., 2022; Lapmanee et al., 2024), haloperidol (Sonego et al., 2018), acute reserpine (Peres et al., 2016) as well as in a transgenic model (Zhao et al., 2022). In these studies, CBD promoted improvements in motor function, memory recognition, metabolism parameters, TH levels, dopaminergic and serotonergic activities, as well as reduction in oxidative stress and inflammatory activity (Garcia et al., 2011; Wang et al., 2022; Lapmanee et al., 2024; Sonego et al., 2018; Zhao et al., 2022). More recently, our group also described the neuroprotective effect of CBD in counteracting reserpine induced behavioral alterations and neuronal degeneration in *Caenorhabditis elegans* (da Cruz Guedes et al., 2023). In another study, based on another feature of PD that is mitochondrial dysfunction (Henrich et al., 2023), CBD increased the lifespan of the animals, and decreased inflammatory markers (such as GFAP and IBA1). These effects could be promoted by PPARγ activation (Puighermanal et al., 2024).

Adding to these robust body of preclinical evidence, our results show the protective impact of CBD administration on the progression of parkinsonian behaviors and on the loss of dopaminergic function in substantia nigra. As mentioned, the preventive administration of CBD (before the induction of the behavioral and dopaminergic abnormalities induced by reserpine) showed more expressive results than concomitant treatment. Reinforcing the more efficient action of the preventive treatment, the CBD/Res group in the concomitant protocol ends the treatment with a catalepsy score similar to the Sal/Res group, which is not the case for the preventive approach. Nevertheless, in both cases, CBD prevents the loss of TH-immunostained cells. This apparent discrepancy could be explained by the fact that the target of CBD action could be multiple (as discussed below), especially considering that the chronic reserpine protocol induces other neuronal alterations beyond the reduction in TH immunostaining, such as oxidative stress, neuroinflammation, alpha-synuclein expression and alteration in other neurotransmitter such as serotonin (Leal et al., 2019). Alternatively, the investigation of TH+ cells across the protocol, at different timepoints, could contribute to clarifying this different magnitude of effects. Further investigation is needed to clarify the mechanisms underlying preventive versus concomitant CBD actions.

In humans, research investigating the neuroprotective effect of CBD is incipient, but a recent systematic review shows that CBD significantly improves parkinsonian symptoms, both acutely and chronically (Bilbao and Spanagel, 2022; de Fatima Dos Santos Sampaio et al., 2024). In addition, some pre-clinical studies and clinical cases demonstrate that a *Cannabis* oil therapy and/or the combination of THC and CBD have positive effects on the treatment of some motor and non-motor symptoms (Peres et al., 2018; de Fatima Dos Santos Sampaio et al., 2024; Lotan et al., 2014; Balash et al., 2017; Yenilmez et al., 2021).

The mechanism of action of CBD is multifaceted (Peres et al., 2018; Alves et al., 2024; Miao et al., 2024). Thus, besides the potential anti-inflammatory and antioxidant actions, the CBD effects in our study could be explained by the influence and/or interaction with other targets, such the modulation of 5-HT1A, promoting an increase in dopamine liberation. Additionally, a direct action in post-synaptic neurons, acting as partial agonist for D2 (Seeman, 2016), and/or the heterodimerization between CB2R and D2 receptors (Alves et al., 2024) can not be ruled out. Finaly, CBD could decrease hyperexcitation by interacting with TRPV1 and GPR55 (Alves et al., 2024); or increasing the synthesis of anti-inflammatory factors by interaction with PPARγ, as mentioned (Puighermanal et al., 2024).

From a clinical point of view, these results reinforce the importance of early diagnosis allowing the use of neuroprotective preventive strategies for the progression of Parkinson’s disease. Although this is still a limitation for the approach studied here, our results point to beneficial effects on in the cases of early diagnosis, suggesting a new possibility to ameliorate PD symptoms or even slowing down the progression of the disease.

With the global population aging and the consequent increase in new cases of Parkinson’s disease, there is a growing demand for new types of pharmacological (Elsworth, 2020; Staats et al., 2023; Zeng et al., 2023) and non-pharmacological treatments (Bloem et al., 2021; Palma and Thijs, 2023). Prevention of disease progression by neuroprotectors arises as a promising strategy (Bloem et al., 2021; Jankovic and Tan, 2020), and the present study reinforces the beneficial profile of early administration (preventive) of CBD.

It is important to mention that this study has some limitations. For example, we only conducted TH+ cells immunostaining at the end of the protocol. Thus, further experimentation is needed to evaluate the effectiveness of CBD on the progression of dopaminergic dysfunction by the inclusion of TH analysis at different time points across the treatment. In addition, the study did not include the analysis of a biomarker for PD. Although the identification of PD biomarkers in patients is still a goal to achieve (Jimenez-Jimenez et al., 2014; Costa et al., 2015), increased expression or aggregation of α-synuclein in animal models can be important evidence of validity (Cannon et al., 2009; Fornai et al., 2005; Yuan et al., 2015), including in the reserpine progressive protocol (Beserra-Filho et al., 2022; Leao et al., 2017; Leao et al., 2021; de Gois et al., 2025).

## 5 Conclusion

The data presented here demonstrate that CBD can attenuate the development of reserpine-induced parkinsonism and protect the loss of dopaminergic neuron in the substantia nigra, with better outcomes in the preventive protocol. The overall effect of CBD is to delay the onset of motor deficits, rather than preventing them entirely. More studies are necessary to understand how CBD exhibits this neuroprotective effect.

## Data Availability

The raw data supporting the conclusions of this article will be made available by the authors, without undue reservation.
