# Supplementary material for: Preventive beneficial effects of cannabidiol in a reserpine-induced progressive model of parkinsonism
Source: Front Pharmacol. 2025 May 8;16:1539783. doi: 10.3389/fphar.2025.1539783 (PMC12095250; doi:10.3389/fphar.2025.1539783)
Supplement: Supplementary file 1 [file Table1.docx]

**Supplementary 1:** (A) Individual data for each animal along the concomitant reserpine protocol. Each phase represents the mean of 5 reserpine applications: day 0 (previously to reserpine administration), premotor (1^st^ to 10^th^ assessments), initial (11^th^ to 20^th^ assessments), intermediate (21^st^ to 30^th^ assessments), and motor (31^st^ to 40^th^ assessments). (B) Mean ± SEM.

**A**

| **#** | **Veh/Res treatment** | **Sal/CBD treatment** | **Day 0** | **Premotor** | **Initial motor** | **Intermediate motor** | **Motor** |
| --- | --- | --- | --- | --- | --- | --- | --- |
| 1 | Veh | Sal | 0.36 | 1.12 | 1.96 | 2.50 | 4.63 |
| 2 | Veh | Sal | 0.52 | 0.61 | 1.06 | 1.06 | 0.92 |
| 3 | Veh | Sal | 0.67 | 0.58 | 2.07 | 4.13 | 5.33 |
| 4 | Veh | Sal | 0.58 | 0.67 | 1.50 | 2.65 | 1.26 |
| 5 | Veh | Sal | 0.47 | 0.65 | 0.93 | 1.30 | 1.28 |
| 6 | Veh | Sal | 0.41 | 1.13 | 1.27 | 3.31 | 2.56 |
| 7 | Veh | Sal | 0.44 | 0.72 | 0.87 | 1.13 | 0.65 |
| 8 | Veh | CBD | 0.54 | 2.76 | 13.53 | 25.52 | 37.48 |
| 9 | Veh | CBD | 0.33 | 1.43 | 2.07 | 2.49 | 2.59 |
| 10 | Veh | CBD | 0.47 | 0.47 | 0.89 | 0.93 | 1.30 |
| 11 | Veh | CBD | 0.49 | 0.54 | 1.40 | 1.44 | 1.22 |
| 12 | Veh | CBD | 0.58 | 0.63 | 1.34 | 1.11 | 1.03 |
| 13 | Veh | CBD | 0.50 | 0.65 | 0.93 | 1.60 | 3.35 |
| 14 | Veh | CBD | 0.54 | 0.57 | 1.12 | 1.41 | 1.03 |
| 15 | Veh | CBD | 0.37 | 2.28 | 12.40 | 15.54 | 27.02 |
| 16 | Veh | CBD | 0.56 | 0.64 | 1.88 | 23.39 | 10.02 |
| 17 | Veh | CBD | 0.45 | 0.92 | 1.40 | 2.64 | 12.61 |
| 18 | Res | Sal | 0.53 | 0.50 | 4.29 | 29.39 | 49.46 |
| 19 | Res | Sal | 0.33 | 0.65 | 57.36 | 119.07 | 146.48 |
| 20 | Res | Sal | 0.39 | 0.57 | 1.58 | 27.10 | 89.86 |
| 21 | Res | Sal | 0.43 | 1.25 | 2.78 | 40.44 | 39.69 |
| 22 | Res | Sal | 0.77 | 0.95 | 0.90 | 9.87 | 29.79 |
| 23 | Res | Sal | 0.54 | 0.87 | 24.22 | 54.07 | 57.01 |
| 24 | Res | Sal | 0.54 | 0.87 | 1.75 | 8.47 | 30.10 |
| 25 | Res | Sal | 0.51 | 0.72 | 3.80 | 60.04 | 49.82 |
| 26 | Res | Sal | 0.66 | 1.92 | 23.36 | 61.68 | 82.98 |
| 27 | Res | Sal | 0.55 | 0.62 | 3.03 | 13.69 | 16.70 |
| 28 | Res | CBD | 0.39 | 0.59 | 1.07 | 11.07 | 31.34 |
| 29 | Res | CBD | 0.45 | 0.52 | 4.00 | 68.40 | 38.69 |
| 30 | Res | CBD | 0.68 | 0.77 | 2.73 | 32.83 | 62.82 |
| 31 | Res | CBD | 0.43 | 1.16 | 4.41 | 66.07 | 120.66 |
| 32 | Res | CBD | 0.53 | 1.19 | 13.29 | 78.37 | 91.68 |
| 33 | Res | CBD | 0.53 | 2.52 | 1.85 | 18.68 | 54.04 |
| 34 | Res | CBD | 0.42 | 0.53 | 7.80 | 31.72 | 61.17 |
| 35 | Res | CBD | 0.62 | 3.89 | 19.26 | 81.31 | 86.49 |
| 36 | Res | CBD | 0.45 | 1.02 | 6.80 | 35.28 | 69.94 |
| 37 | Res | CBD | 0.34 | 0.52 | 2.58 | 10.83 | 14.29 |

**B**

|  |  | **Day 0** | **Premotor** | **Initial motor** | **Intermediate motor** | **Motor** |
| --- | --- | --- | --- | --- | --- | --- |
| Veh | Sal | 0.49 ±0.04 | 0.78 ±0.09 | 1.38 ±0.18 | 2.29 ±0.44 | 2.38 ±0.71 |
|  | CBD | 0.48 ±0.02 | 1.09 ±0.25 | 3.70 ±1.55 | 7.61 ±3.13 | 9.76 ±4.03 |
| Res | Sal | 0.53 ±0.04 | 0.89 ±0.13 | 12.31 ±5.73 | 42.38 ±10.61 | 59.19 ±12.11 |
|  | CBD | 0.48 ±0.03 | 1.27 ±0.34 | 6.38 ±1.83 | 43.46 ±8.71 | 63.11 ±9.88 |

**Supplementary 2:** (A) Individual data for each animal along the preventive reserpine protocol. Each phase represents the mean of 5 reserpine applications: pre reserpine and day 0 (previously to reserpine administration), premotor (1^st^ to 10^th^ assessments), initial (11^th^ to 20^th^ assessments), intermediate (21^st^ to 30^th^ assessments), and motor (31^st^ to 40^th^ assessments). (B) Mean ± SEM.

**A**

| **#** | **Veh/Res treatment** | **Sal/CBD treatment** | **Pre reserpine** | **Day 0** | **Premotor** | **Initiate motor** | **Intermediate motor** | **Motor** |
| --- | --- | --- | --- | --- | --- | --- | --- | --- |
| 1 | Veh | Sal | 0.45 | 0.55 | 0.69 | 2.17 | 1.70 | 1.66 |
| 2 | Veh | Sal | 0.31 | 0.30 | 0.36 | 0.35 | 0.53 | 0.48 |
| 3 | Veh | Sal | 0.52 | 0.36 | 0.37 | 2.20 | 2.60 | 3.66 |
| 4 | Veh | Sal | 0.33 | 0.37 | 0.44 | 0.92 | 1.62 | 3.96 |
| 5 | Veh | Sal | 0.34 | 0.29 | 0.34 | 0.44 | 0.80 | 0.56 |
| 6 | Veh | Sal | 0.39 | 0.32 | 0.55 | 0.55 | 0.58 | 1.08 |
| 7 | Veh | Sal | 0.27 | 0.27 | 0.43 | 0.47 | 0.56 | 0.68 |
| 8 | Veh | Sal | 0.43 | 0.38 | 0.58 | 1.67 | 2.64 | 2.67 |
| 9 | Veh | Sal | 0.35 | 0.46 | 0.58 | 1.10 | 0.63 | 0.87 |
| 10 | Veh | CBD | 0.58 | 0.33 | 1.00 | 0.86 | 1.66 | 4.06 |
| 11 | Veh | CBD | 0.36 | 0.40 | 0.41 | 0.45 | 0.93 | 1.93 |
| 12 | Veh | CBD | 0.42 | 0.48 | 0.40 | 0.62 | 1.07 | 0.69 |
| 13 | Veh | CBD | 0.28 | 0.28 | 0.32 | 0.36 | 0.47 | 0.59 |
| 14 | Veh | CBD | 0.34 | 0.32 | 0.34 | 0.37 | 0.36 | 0.50 |
| 15 | Veh | CBD | 0.30 | 0.25 | 0.33 | 0.38 | 0.37 | 0.37 |
| 16 | Veh | CBD | 0.32 | 0.35 | 0.46 | 0.68 | 0.61 | 0.57 |
| 17 | Veh | CBD | 0.39 | 1.29 | 0.87 | 1.15 | 0.90 | 1.43 |
| 18 | Veh | CBD | 0.61 | 0.48 | 1.41 | 0.78 | 2.86 | 0.61 |
| 19 | Res | Sal | 0.36 | 0.29 | 0.39 | 0.57 | 2.79 | 14.74 |
| 20 | Res | Sal | 0.78 | 0.36 | 0.69 | 6.58 | 24.62 | 88.68 |
| 21 | Res | Sal | 0.38 | 0.55 | 0.63 | 10.12 | 30.79 | 66.60 |
| 22 | Res | Sal | 0.32 | 0.36 | 0.40 | 1.22 | 11.71 | 19.69 |
| 23 | Res | Sal | 0.31 | 0.31 | 0.34 | 0.65 | 5.14 | 21.93 |
| 24 | Res | Sal | 0.28 | 0.28 | 0.93 | 1.31 | 1.92 | 6.49 |
| 25 | Res | Sal | 0.29 | 0.29 | 0.50 | 0.55 | 0.76 | 0.78 |
| 26 | Res | Sal | 0.37 | 0.77 | 1.10 | 1.44 | 7.16 | 18.13 |
| 27 | Res | Sal | 0.29 | 0.34 | 0.38 | 1.76 | 1.50 | 8.90 |
| 28 | Res | Sal | 0.51 | 3.04 | 7.12 | 17.47 | 24.15 | 40.59 |
| 29 | Res | CBD | 0.30 | 0.31 | 0.32 | 0.45 | 0.73 | 1.53 |
| 30 | Res | CBD | 0.34 | 0.48 | 0.54 | 1.69 | 4.66 | 16.92 |
| 31 | Res | CBD | 0.38 | 0.33 | 0.42 | 1.33 | 19.80 | 80.86 |
| 32 | Res | CBD | 0.37 | 0.52 | 0.68 | 1.73 | 6.50 | 30.10 |
| 33 | Res | CBD | 0.32 | 0.42 | 0.36 | 0.44 | 3.98 | 21.45 |
| 34 | Res | CBD | 0.33 | 0.33 | 0.67 | 0.80 | 1.25 | 2.78 |
| 35 | Res | CBD | 0.32 | 0.33 | 0.43 | 0.58 | 0.62 | 0.61 |
| 36 | Res | CBD | 0.36 | 0.27 | 0.49 | 0.82 | 2.37 | 2.50 |
| 37 | Res | CBD | 0.44 | 0.49 | 1.02 | 2.98 | 10.82 | 22.42 |
| 38 | Res | CBD | 0.32 | 0.44 | 0.60 | 0.79 | 0.71 | 0.88 |

**B**

|  |  | **Pre reserpine** | **Day 0** | **Premotor** | **Initial motor** | **Intermediate motor** | **Motor** |
| --- | --- | --- | --- | --- | --- | --- | --- |
| Veh | Sal | 0.38 ±0.02 | 0.37 ±0.02 | 0.48 ±0.04 | 1.10 ±0.24 | 1.30 ±0.29 | 1.74 ±0.45 |
|  | CBD | 0.40 ±0.03 | 0.46 ±0.10 | 0.62 ±0.12 | 0.63 ±0.08 | 1.02 ±0.26 | 1.19 ±0.39 |
| Res | Sal | 0.39 ±0.04 | 0.66 ±0.26 | 1.25 ±0.65 | 4.17 ±1.78 | 11.05 ±3.56 | 28.65 ±8.98 |
|  | CBD | 0.35 ±0.01 | 0.39 ±0.02 | 0.55 ±0.06 | 1.16 ±0.25 | 5.14 ±1.92 | 18.00 ±7.79 |
